# Causal relationship between 150 skin microbiomes and prostate cancer: insights from bidirectional mendelian randomization and meta-analysis

**DOI:** 10.3389/fimmu.2024.1463309

**Published:** 2024-09-25

**Authors:** Daolei Chen, Songqi Hu, Xinchao Wang, Zhisi Chen, Wanxian Xu

**Affiliations:** ^1^ Department of Surgery, First People’s Hospital of Kunming City & Calmette Affiliated Hospital of Kunming Medical University, Kunming, Yunnan, China; ^2^ Graduate School, Kunming Medical University, Kunming, China

**Keywords:** skin microbiomes, prostate cancer, bidirectional Mendelian randomization, meta-analysis, genome-wide association study

## Abstract

**Background:**

Despite relevant research, the relationship between skin microbiomes and prostate cancer remains controversial. This study utilizes bidirectional Mendelian randomization (MR) analysis combined with meta-analysis to explore the potential link between the two.

**Objective:**

This study aims to identify the causal relationship between 150 skin microbiomes and prostate cancer (PCa) using bidirectional Mendelian randomization (MR) and meta-analysis.

**Methods:**

This study employed a comprehensive Bidirectional Two-sample MR analysis using publicly available genetic data to ascertain the relationship between 150 skin microbiomes and PCa. We conducted extensive sensitivity analyses, tests for heterogeneity, and assessments of horizontal pleiotropy to ensure the accuracy of our results. Subsequently, we conducted a meta-analysis to strengthen our conclusions’ robustness further. Finally, we performed reverse causal verification on the positive skin microbiomes and PCa.

**Results:**

After conducting a meta-analysis and multiple corrections of the MR analysis results, our findings reveal a correlation between Neisseria in dry skin and PCa risk, identifying it as a risk factor. The IVW result shows an *Odds Ratio (OR)* of 1.009 (95% *Confidence Interval [CI]*: 1.004-1.014, *P* = 0.027). Furthermore, the reverse MR analysis indicates the absence of an inverse causal relationship between the two. Apart from the identified skin microbiome, no significant associations were found between the other microbiomes and PCa.

**Conclusions:**

The study identified a correlation between Neisseria in dry skin, one of the 150 skin microbiomes, and the risk of developing PCa, establishing it as a risk factor for increased susceptibility to PCa.

## 1 Introduction

Prostate cancer (PCa) is one of the most common malignant tumors among men worldwide, with approximately 1.5 million new cases diagnosed each year, accounting for 7.3% of all new cancer cases globally (1). This makes it the second most common cancer type among men, second only to lung cancer (2, 3). The exact etiology of PCa is not fully understood. Current perspectives suggest that genetic factors, racial factors, lifestyle, and dietary habits are all risk factors for the development of PCa (4). Additionally, age is a significant risk factor for PCa (5), with the incidence rate remaining high due to the increasing aging population, making PCa a critical factor affecting men’s physical and mental health globally.

Although early detection and treatment can significantly improve cure rates, with the five-year survival rate for early PCa approaching 100% (6), the prognosis for advanced PCa is poor, with a five-year survival rate of approximately 30% (5). Advanced patients often suffer from severe complications, including urinary incontinence (7), sexual dysfunction (8), bone pain due to bone metastases (9), and pathological fractures (9), severely impacting their physical and mental well-being. Given the high incidence and complication risks, in-depth research on the etiology, prevention, early screening, and treatment of PCa is crucial to optimizing public health strategies, increasing early detection rates, and improving patients’ quality of life, thereby effectively addressing this global health challenge.

The skin is the largest organ of the human body, with a surface area of approximately 1.8 square meters, hosting a rich and complex microbiome (10). These microbiomes mainly include bacteria, fungi, viruses, and parasites, with bacteria being the predominant group (11). The composition and diversity of skin microbiomes are influenced by various factors, including the host’s age, gender, ethnicity, geographic location, lifestyle, immune status, and disease state (12). Skin microbiomes play crucial roles in maintaining skin barrier function, regulating immune responses, and preventing pathogenic infections (13). Recent research has increasingly shown that dysbiosis of the skin microbiome may be closely related to various skin diseases and systemic conditions (11, 14).

In recent years, research on the relationship between microbiomes and cancer has gradually increased. The relationship between gut microbiota and colorectal cancer has been extensively studied and recognized (15). Both gut and skin microbiota play important roles in regulating host health, with interactions between them potentially coordinated through the “gut-skin axis,” thereby regulating host health by modulating the immune system and metabolic pathways (16). Previous studies have suggested that the skin microbiome might influence cancer development and progression through various mechanisms (17). Consistent findings indicate a close relationship between the skin microbiome and certain malignancies, such as leukemia (18).

Although studies on the relationship between PCa and the skin microbiome are relatively limited, existing research indicates significant changes in the composition and diversity of the microbiome in PCa patients, suggesting that microbiomes, including those of the skin, may play important roles in the development and progression of PCa (19). This underscores the importance of studying the relationship between the skin microbiome and PCa for the diagnosis and development of new therapeutic targets. Moreover, a study by Davidsson et al. found that Propionibacterium acnes in the skin of patients with prostatitis might be associated with an increased risk of PCa, highlighting the close relationship and significant research value of skin microbiome alterations in PCa (20). However, due to confounding factors and reverse causality, traditional observational studies cannot fully elucidate the relationship between various skin microbiomes and PCa. Additionally, conducting randomized controlled trials to explore this relationship faces significant challenges, including sample size, funding, time constraints, and ethical considerations.

Over the past two decades, MR has gained attention as a reliable research method (21). It uses genetic variants, such as single nucleotide polymorphisms (SNPs), as instrumental variables (IVs) to establish causal relationships between exposure factors and disease outcomes. These variants are associated with the exposure of interest but are not influenced by lifestyle or socioeconomic factors. This method reduces the impact of confounding factors and reverses causality common in traditional observational studies, thereby enhancing the robustness of the results. IVs used in MR studies must meet three key assumptions: they should be strongly associated with the intermediate exposure, independent of confounding factors, and not exert direct pleiotropic effects on the outcome. These assumptions make MR a powerful method for establishing causal relationships in the absence of randomized controlled trials.

Unlike traditional unidirectional MR, bidirectional two-sample MR analysis allows researchers to explore causal relationships in both directions (22). This helps rule out reverse causality and enhances the credibility of the findings by verifying bidirectional causality. Significant causal relationships found in both directions suggest a more likely true association rather than one caused by confounding factors or biases. Additionally, this method leverages genetic and phenotypic data from different datasets, expanding the study scope and enhancing the generalizability of the findings.

Therefore, this study employs bidirectional two-sample MR analysis to explore the causal relationships between 150 skin microbiome phenotypes and PCa, elucidating both forward and reverse associations. We further conducted a meta-analysis on the PCa results from different databases to strengthen the robustness of our findings. After completing the MR analysis, we applied the Bonferroni correction to account for multiple testing. Finally, we performed reverse MR analysis to verify the reverse causal relationship between the skin microbiome phenotypes and PCa phenotypes that showed positive outcomes, thereby excluding the impact of reverse causality and enhancing the robustness of our results.

## 2 Materials and methods

### 2.1 Study design

**Figure 1:**
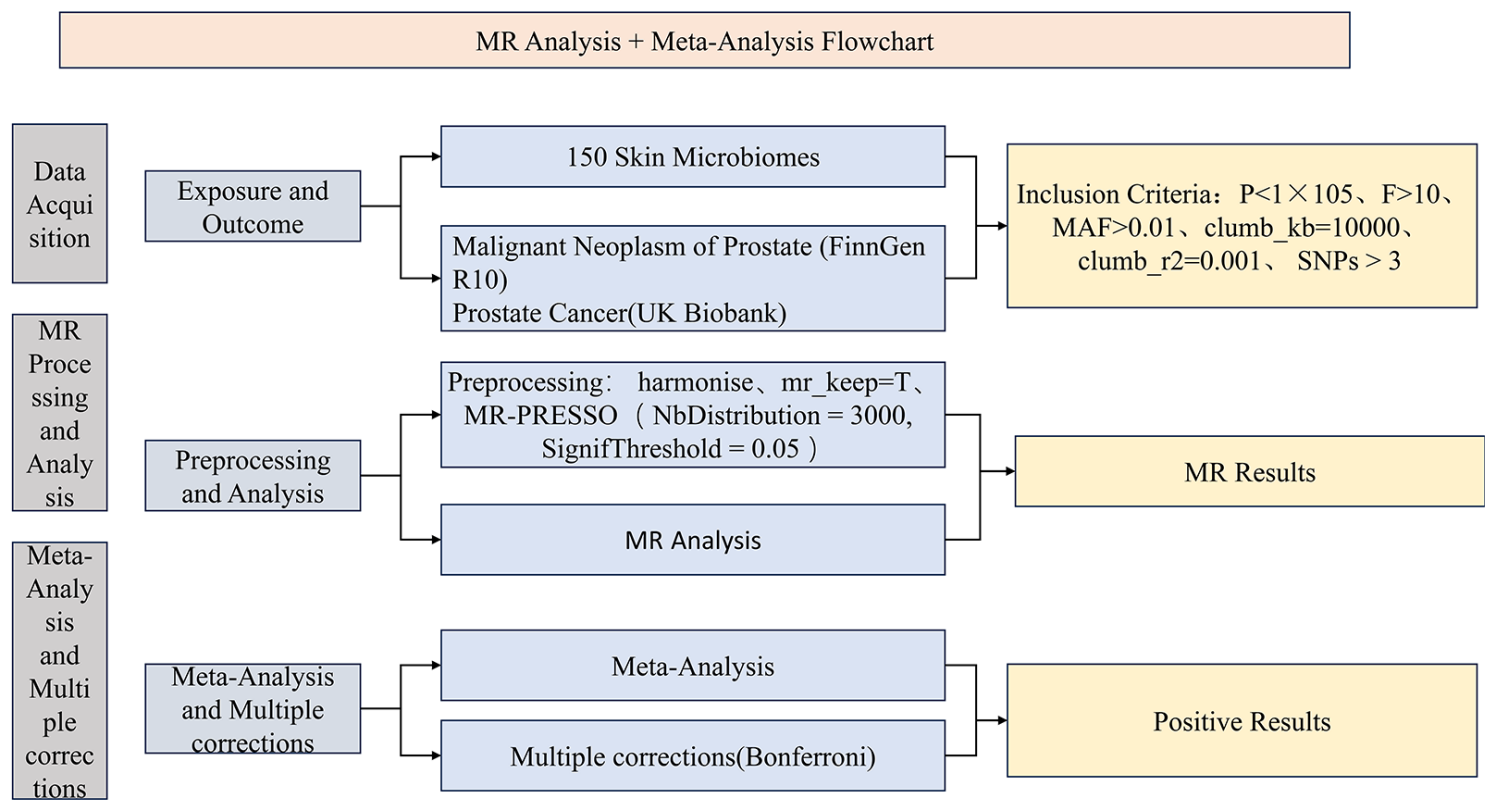
The process flowchart of the research methodology.

During the study, we first collected optimal exposure and outcome data and performed data preprocessing. Subsequently, we conducted MR analysis using the preprocessed exposure data (150 skin microbiomes) against outcome data (PCa) from two distinct databases. We then performed a meta-analysis (23) on the IVW results derived from MR analyses using PCa data from different databases and conducted multiple corrections on the meta-analysis results to ensure data accuracy. Finally, we designated the skin microbiomes identified as positive outcomes as the exposure data and PCa as the outcome data, employing the same instrumental variable selection and data analysis methods as in the forward analysis to perform a reverse MR analysis. The advantage of combining MR analysis with meta-analysis lies in its ability to synthesize results from multiple studies, reduce result bias, explore heterogeneity, and enhance the generalizability of the findings. By integrating data from different studies, we can more comprehensively assess the association between exposure and outcome, providing more reliable results and deepening our understanding of the research question. This study utilizes large public datasets, all of which have received approval from relevant institutions and associations, and informed consent was obtained from all participants. Consequently, no additional ethical review is required for this study. To present the research process more clearly, we have drawn a corresponding flowchart (**Figure 1**). Additionally, our study confirms adherence to the STROBE-MR guidelines (**Supplementary Table 1**).

### 2.2 Sources of data for the GWAS on 150 skin microbiomes

The data on the 150 types of skin microbiota used in this study were sourced from the research conducted by Moitinho-Silva et al., published in 2022 in Nature Communications, titled “Host genetic factors related to innate immunity, environmental sensing, and cellular functions are associated with human skin microbiota.” This study performed genome-wide association studies (GWAS) on two German cohorts, including 474 cases and 419 controls, totaling 893 samples. It analyzed the microbiota from different skin microenvironments (dry skin, moist skin, and sebaceous-rich skin), specifically measuring 837,260 SNP markers. These SNP markers were used in GWAS to identify host genetic factors associated with skin microbiota. The study involved 150 different skin microbiota traits and revealed 23 genetic loci significantly associated with skin microbiota characteristics. These genes are mainly involved in innate immune signaling, environmental sensing, cellular differentiation, and proliferation. The data have been included in the public database GWAS Catalog, with the registration numbers GCST90133164-GCST90133313. We downloaded the manually curated complete data from the GWAS Catalog database (download link: https://www.ebi.ac.uk/gwas/publications/36261456, download date: June 1, 2024).

### 2.3 Sources of data for the GWAS on PCa

The PCa data were aggregated from two GWAS datasets involving European populations. FinnGen is a large public-private partnership project that utilizes Finland’s unique genetic resources to generate high-quality genetic and health data. This project combines whole-genome data from approximately 500,000 Finnish individuals with extensive health registry information, representing nearly 10% of Finland’s total population. The first dataset (referred to as FinnGen) is derived from the R10 release of the FinnGen database (https://www.finngen.fi/en), encompassing a GWAS on146,465 Europeans (15,199 cases, 131,266 controls) (24). The UK Biobank (UKB) is a significant biomedical research resource, supported by the UK government, the Medical Research Council (MRC), and the Wellcome Trust. This database includes detailed health and genetic information from around 500,000 UK participants aged 40 to 69, covering various traits and diseases. Users can easily access the required data through search and download functionalities, supporting genetic analyses and causal inference studies. The UK Biobank aims to promote open data sharing, advancing genetic epidemiology and public health research, making it a crucial tool for global medical research. The second dataset (referred to as UKB) is sourced from the UK Biobank (Pan-UKB team. https://pan.ukbb.broadinstitute.org.2020.), including a GWAS on 364,233 Europeans (28,010 cases, 336,223 controls). Both datasets have undergone manual review to ensure data accuracy.

## 3 Statistical analysis

### 3.1 Selection and Standardization of IVs

In omics MR studies, selecting effective instrumental variables (IVs) is crucial. Initially, this study employed a selection threshold of *P* < 1x10^-5^ to ensure that only SNPs strongly associated with various micronutrients were retained. For most micronutrients, the number of associated SNPs exceeded three, ensuring the representativeness and relevance of the data. Next, to further refine the selection of robust instrumental variables, the F-statistic for each SNP was calculated using the formula F = (beta/se) ². Only SNPs with an F value greater than 10 were retained, a step that helps eliminate weak instrumental variables and enhances the reliability of the study’s findings. Additionally, the minor allele frequency (MAF) was calculated using the effect allele frequency (eaf). If the eaf was less than 0.5, the MAF was set to eaf; otherwise, it was set to 1 - eaf. Only SNPs with an MAF greater than 0.01 were retained to exclude rare variants that could affect the study’s results. Finally, the filtered data were formatted for MR analysis, and linkage disequilibrium (LD) was addressed to avoid its impact on the accuracy of the results. Specifically, the distance threshold was set at 10,000 kilobases (kb) and the LD threshold at 0.001. These steps ensured the independence of the instrumental variables and the precision of the results (The results are shown in **Supplementary Table 1**). Overall, the specific selection criteria were: *P* < 1x10^-5^, *MAF* > 0.01, *F* > 10, *clump_kb* = 10000, and *clump_r2 = *0.001.

### 3.2 Mendelian randomization

In the first step of the forward analysis, we extracted SNP data from the PCa phenotype outcome that matches the 150 skin microbiome phenotypes. Next, we processed the palindromic SNPs based on the specific criterion of action = 2. Additionally, we excluded data with mr_keep = false.

Before performing MR-PRESSO, we conducted a horizontal pleiotropy test on the processed data. If an SNP had a p-value less than 0.05, it was considered horizontally pleiotropic, indicating an outlier, and subsequently removed using the MR-PRESSO method to ensure data accuracy. The specific parameters for MR-PRESSO were set to NbDistribution = 3000 and SignifThreshold = 0.05. We then excluded SNPs with p-values less than 0.05.

After meticulously processing the data and before proceeding with MR analysis, we also performed a heterogeneity test. Although data heterogeneity has minimal impact on the results, we used the IVW random effects model for MR analysis on heterogeneous SNPs (Q_pval < 0.05) and the IVW fixed effects model for non-heterogeneous SNPs. Additionally, regardless of the presence of heterogeneity, we analyzed the data using the MR-Egger and weighted median methods and calculated their OR values. To enhance the reliability of the results, we conducted a meta-analysis of the MR results for the 150 skin microbiome phenotypes with the two PCa groups and applied multiple corrections to the meta-analysis significance p-values using the Bonferroni correction method to reduce the likelihood of Type I errors.

### 3.3 Sensitivity analyses

Horizontal pleiotropy means that different treatments or interventions may have varying effects on different individuals or contexts, which could be mistakenly attributed to differences between the experimental and control groups rather than the actual treatment effect (25). To minimize the impact of horizontal pleiotropy on experimental results, we conducted horizontal pleiotropy testing on the GWAS data. We used MR-PRESSO to exclude SNPs exhibiting horizontal pleiotropy (*P* < 0.05), with the exclusion criteria set as NbDistribution = 3000 and SignifThreshold = 0.05. Heterogeneity refers to the diversity or variability among study subjects, observations, or experimental conditions (26). In statistics and research methodology, heterogeneity typically denotes differences among samples or individuals, which may stem from individual characteristics and environmental factors, including physiological and psychological conditions or other influences. Common forms of heterogeneity in research may manifest as physiological differences among individuals, variations in socioeconomic status, and the effects of environmental factors. This diversity and variability enhance the generalizability and representativeness of the research findings but also increase the complexity and difficulty of interpretation. During the analysis, we also conducted heterogeneity testing on the data. For SNPs exhibiting heterogeneity (*Q-pval* < 0.05), we performed MR analysis using a random-effects model within the IVW method. Otherwise, we employed a fixed-effects model to ensure the accuracy and reliability of the results (27).

### 3.4 Bonferroni correction and meta-analysis

The research process involved conducting MR analyses to assess the association between exposure and outcomes from two different sources, followed by a meta-analysis of the MR results and multiple corrections. Specifically, the study analyzed the relationship between 150 skin microbiome phenotypes and prostate cancer, utilizing MR analysis results from the UKB and FinnGen databases. The most significant IVW results from the MR analyses were subsequently subjected to meta-analysis using the meta package in R. We then applied the Bonferroni method for multiple corrections to the significant P-values from the meta-analysis to reduce the occurrence of Type I errors (28).

The meta-analysis approach has been previously developed and applied in MR research. For instance, a Mendelian Randomization study by Noordam et al. on the association between circulating antioxidants and the risk of coronary heart disease provided a detailed account of the MR analysis process using data on coronary heart disease outcomes from three different databases. The IVW results from these three analyses were subsequently meta-analyzed, and the final results did not support the hypothesis that circulating antioxidants have a protective effect against coronary heart disease (23).

### 3.5 Ascertainment of positive outcomes and reverse MR analysis

Our positive results must meet the following criteria: 1) The meta-analysis results using the IVW method have an adjusted P-value less than 0.05 after multiple corrections; 2) The results of the IVW, weighted median, and MR-Egger methods are consistent in direction (same sign of β value); 3) There is no evidence of horizontal pleiotropy or heterogeneity. Subsequently, we used the skin microbiomes that yielded positive results as the outcomes and performed reverse analysis using PCa phenotypes from different sources as the exposures to test for potential reverse causality between them. By reducing the interference of reverse causality, this approach allows us to more accurately determine the direction of the causal relationship, thereby enhancing the credibility of the forward MR analysis.

## 4 Results

### 4.1 The influence of 150 skin microbiomes on PCa

**Figure 2:**
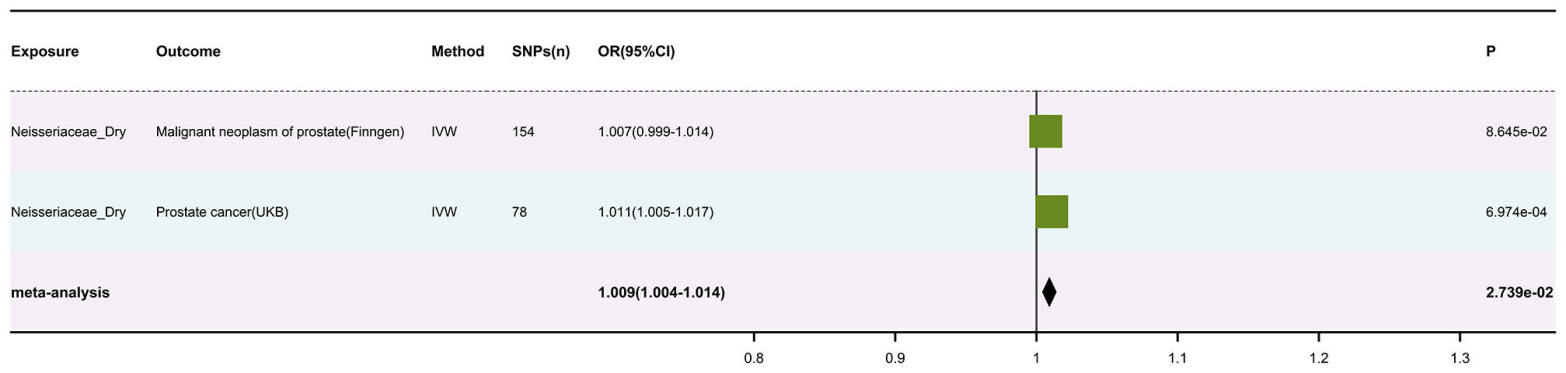
MR scatter diagram of neisseriaceae_dry levels’ impact on PCa phenotype from FinGen data.

After meticulous analysis and processing of the data, we identified 12,251 usable SNPs (results in **Supplementary Table 2**). Subsequently, we conducted an MR analysis using these SNPs for 150 skin microbiota and two sets of skin cancer data (results in **Supplementary Table 3**). A meta-analysis of the IVW results from the MR analysis was performed (results in **Supplementary Table 4**), with multiple corrections applied to the P values of the meta-analysis. Ultimately, we found a significant association between Neisseriaceae_Dry (GWAS ID: GCST90133217) and skin cancer, with Neisseriaceae_Dry acting as a protective factor (The visualized forest plot of the results is shown in **Figure 2**, and detailed results can be found in **Supplementary Table 5**).

It is noteworthy that among all the MR analyses and subsequent meta-analyses of skin microbiomes, eight taxa showed significant causal associations. Specifically, the *P-value* for Acinetobacter_Dry in the combined MR and meta-analysis was 0.045, ASV042_Dry had a *P-value* of 0.047, Bacteroides_Moist had a *P-value* of 0.020, Clostridiales_Incertae_Sedis_XI_Dry had a *P-value* of 0.028, Gammaproteobacteria_Moist had a *P-value* of 0.025, Neisseriaceae_Dry had a *P-value* of 0.0002, Rhodobacteraceae_Moist had a *P-value* of 0.046, and Streptococcus_Moist had a *P-value* of 0.0085. However, since these results had not been subjected to multiple corrections, the false positive rate could be significantly increased. Therefore, we applied Bonferroni correction to the above results. After correction, only Neisseriaceae_Dry showed a strong significant causal association, with a corrected *P-value* of 0.016.

**Figure 3:**
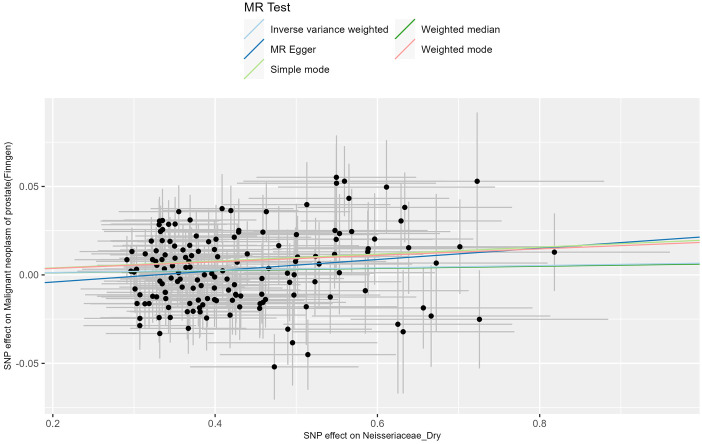
MR scatter diagram of neisseriaceae_dry levels’ impact on PCa phenotype from UKB data.

The IVW results of the MR analysis for the prostate cancer phenotype in the FinnGen database with Neisseriaceae_Dry show an *OR* of 1.007 (95% *CI*: 0.999-1.014, *P* = 0.086). The scatter plot of the MR results is displayed in **Figure 3**, with the most strongly significant SNPs being: rs10516313, rs13075225, rs17605241, rs2095502, rs2422596, rs2731840, rs3017687, rs6025143, rs7143789, rs7512417, rs80196090 (detailed information can be found in **Table 1**). For the MR results between the prostate cancer phenotype in the FinnGen database and Neisseriaceae bacteria, the IVW method, MR-Egger method, and weighted median method all yielded significant positive *β* values and *OR* values greater than 1, suggesting that this trait may increase the risk of prostate cancer.

**Table 1:** Strongly significant SNPs in the MR effect of neisseriaceae_dry bacteria on prostate cancer phenotype in the FinnGen database.

SNP	effect_allele.exposure	other_allele.exposure	effect_allele.outcome	other_allele.outcome	R^2^	F.value
rs10516313	T	C	T	C	0.069	30.128
rs13075225	T	C	T	C	0.075	32.747
rs17605241	A	G	A	G	0.071	31.037
rs2095502	A	G	A	G	0.063	27.228
rs2422596	A	C	A	C	0.056	23.857
rs2731840	T	C	T	C	0.062	26.552
rs3017687	A	G	A	G	0.060	25.819
rs6025143	A	C	A	C	0.058	24.855
rs7143789	A	G	A	G	0.062	26.796
rs7512417	A	G	A	G	0.072	31.332
rs80196090	T	C	T	C	0.079	34.555

**Figure 4:**
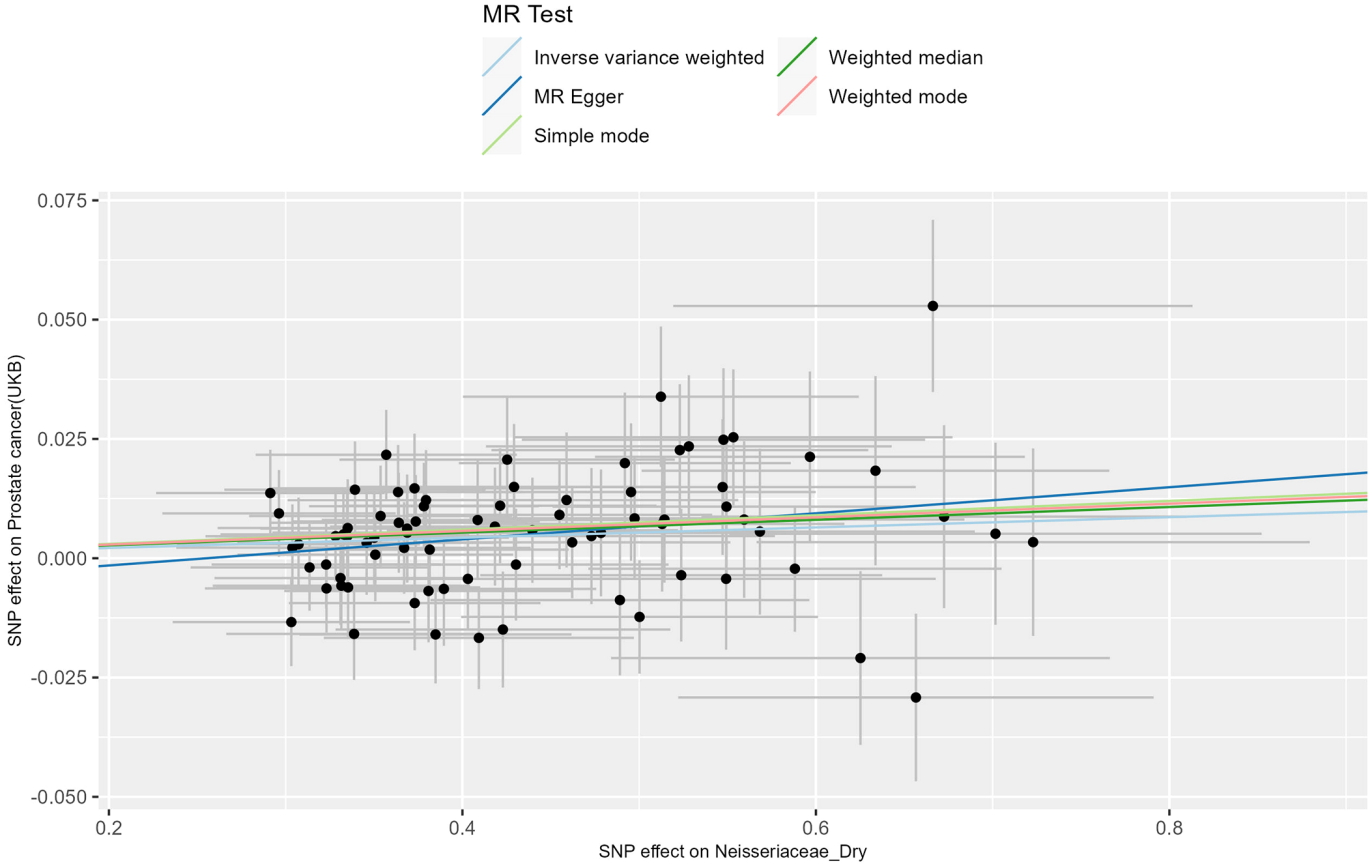
Forest plot of positive results after meta-analysis.

The MR analysis of the prostate cancer phenotype in the UKB database with Neisseriaceae_Dry shows an *OR* of 1.011 (95% *CI*: 1.005-1.017, *P* = 0.001). The scatter plot of the MR results is displayed in **Figure 4**, with the most strongly significant SNPs being: rs10516313, rs11699213, rs12136471, rs13075225, rs17605241, rs2095502, rs3017687, rs4719814, rs476951, rs7143789, rs74443042, and rs7512417 (detailed information can be found in **Table 2**). For the MR results between the prostate cancer phenotype in the UKB database and Neisseriaceae bacteria, the IVW method, MR-Egger method, and weighted median method all yielded significant positive β values and OR values greater than 1, similarly indicating that this trait may increase the risk of prostate cancer.

**Table 2:** Strongly significant SNPs in the MR effect of neisseriaceae_dry bacteria on prostate cancer phenotype in the UKB database.

SNP	effect_allele.exposure	other_allele.exposure	effect_allele.outcome	other_allele.outcome	R2	F.value
rs10516313	T	C	T	C	0.069	30.128
rs11699213	T	G	T	G	0.067	28.784
rs12136471	T	C	T	C	0.060	25.581
rs13075225	T	C	T	C	0.075	32.747
rs17605241	A	G	A	G	0.071	31.037
rs2095502	A	G	A	G	0.063	27.228
rs3017687	A	G	A	G	0.060	25.819
rs4719814	A	G	A	G	0.062	26.633
rs476951	A	G	A	G	0.065	28.007
rs7143789	A	G	A	G	0.062	26.796
rs74443042	A	G	A	G	0.057	24.565
rs7512417	A	G	A	G	0.072	31.332

Subsequently, a meta-analysis of the results from these two outcome databases was conducted, and the *P-values* from the meta-analysis were corrected for multiple comparisons. The results showed an *OR* of 1.009 (95% *CI*: 1.004-1.014, *P* = 0.027), with the forest plot visualizing the results shown in **Figure 2**. Moreover, the analysis results of Neisseriaceae bacteria in the FinnGen and UK Biobank databases indicate a positive correlation with prostate cancer.

### 4.2 Sensitivity analysis results

For the sensitivity analysis results of the positive outcomes, Cochran’s Q test did not reveal a *Q_pval* < 0.05, indicating no significant heterogeneity in our results (**Supplementary Table 6**). Additionally, in the above results, for SNPs with horizontal pleiotropy, the MR-PRESSO method removed outliers, and the results are shown in **Supplementary Table 7**.

### 4.3 The Influence of PCa on the positive micronutrients

We used reverse MR analysis to assess the causal effect of PCa on Neisseriaceae_Dry. The primary analytical methods included IVW, MR Egger, and weighted median methods to ensure the robustness and reliability of the results. Using Finngen R10 data, the IVW method showed no significant causal relationship between PCa and the abundance of Neisseriaceae (*β* = -0.096, *SE* = 0.305, *P* = 0.752, *OR* = 0.91, 95% *CI* = 0.50 - 1.65). The MR Egger analysis also showed no significant causal relationship between PCa and Neisseriaceae abundance *(β* = 0.007, *SE* = 1.170, *P* = 0.995, *OR* = 1.01, 95% *CI* = 0.10 - 9.99). The weighted median method indicated no significant causal relationship of PCa on Neisseriaceae abundance (*β* = -0.435, *SE* = 0.280, *P* = 0.121, *OR* = 0.65, 95% *CI* = 0.37 - 1.12). Using UKB data, the IVW method again showed no significant causal relationship between PCa and the abundance of Neisseriaceae (*β* = -0.072, *SE* = 0.504, P = 0.886, *OR* = 0.93, 95% *CI* = 0.35 - 2.50). The MR Egger analysis showed no significant causal relationship between PCa and Neisseriaceae abundance (*β* = 0.156, *SE* = 2.284, *P* = 0.948, *OR* = 1.17, 95% *CI* = 0.01 - 102.80). These results suggest that across the different statistical methods and datasets used, PCa does not have a significant causal effect on Neisseriaceae_Dry levels. (**Supplementary Table 8**).

## 5 Discussion

In this study, we found a significant positive correlation between Neisseriaceae bacteria in dry skin and prostate cancer (PCa) through bidirectional MR analysis combined with meta-analysis. Specifically, the analysis results from the FinnGen and UKB databases showed that exposure to this bacterium was associated with an increased risk of prostate cancer. This association remained statistically significant after meta-analysis and Bonferroni correction.

Notably, only Neisseriaceae bacteria in dry skin environments showed a positive correlation with prostate cancer, whereas in moist and sebaceous gland environments, Neisseriaceae bacteria exhibited negative results. We hypothesize that the microbial composition of dry skin environments differs from that of moist or sebaceous gland-rich environments. The dry environment may promote the growth and proliferation of certain Neisseriaceae bacteria, making them more likely to interact with host cells and influence disease risk (12, 13). In moist or sebaceous gland-rich environments, other microorganisms may dominate, inhibiting the growth of Neisseriaceae bacteria (11). The diverse microbiome in these environments may limit the proliferation of Neisseriaceae bacteria and their potential pathogenic effects through competitive mechanisms (29).

Neisseriaceae bacteria in dry skin may influence the development of PCa through several potential biological mechanisms. Firstly, the regulation of the immune system may play a crucial role (29). Neisseriaceae bacteria in dry skin may affect local and systemic immune responses, stimulating immune cells in the skin and inducing the production of various cytokines and inflammatory mediators (30). These immune responses may further impact the overall immune status, including the prostate. Chronic low-grade inflammation over time may promote malignant transformation in prostate tissue (31, 32). Neisseriaceae bacteria may induce chronic inflammation in the skin and systemically through their metabolites or direct bacteria-cell interactions (17).

Secondly, the interaction between the microbiome and the endocrine system is also an important consideration. Skin microbiota may influence the endocrine system through complex pathways (33). Bacteria may be involved in regulating hormone levels in the skin and throughout the body (16). It is known that prostate cancer is closely related to androgen levels (e.g., testosterone) (34), and skin bacteria may indirectly affect the hormonal environment of the prostate. Additionally, the skin’s bacterial community may influence the metabolism of corticosteroids, which help regulate immune and inflammatory responses, thereby indirectly affecting the prostate’s health (17).

Bacterial metabolites may also play a role in this process. Neisseriaceae bacteria may produce certain metabolites that can reach the prostate through the bloodstream and have direct pro-cancer or anti-cancer effects on its cells (35, 36). For example, certain lipids or short-chain fatty acids may have pro-cancer effects (37). Furthermore, microorganisms may compete with host cells for certain nutrients or metabolites, and the deficiency or excess of these substances may affect the growth and proliferation of prostate cells (38).

Finally, gene-environment interactions also need to be considered. Host genes may determine individual susceptibility to Neisseriaceae bacteria and the composition of the microbiome (39, 40). These genetic factors may also simultaneously influence the risk of prostate cancer. Gene-environment interactions establish a link between these two factors.

Our research process involved multi-center database validation, confirming the robustness of the findings with consistent results across different databases (FinnGen and UKB). Meta-analysis and Bonferroni correction were used to ensure statistical significance, reducing the likelihood of false positives and obtaining reliable research results. However, further experimental and biological studies are necessary to validate the conclusions, including *in vitro* cell experiments, animal models, and clinical studies. Our study provides a foundation and direction for understanding and further exploring the relationship between Neisseriaceae bacteria in dry skin and prostate cancer. Through bidirectional MR analysis combined with meta-analysis, we found a significant positive correlation between Neisseriaceae bacteria in dry skin and PCa.

The study reveals a potential mechanism linking Neisseriaceae bacteria in dry skin to a significant positive association with prostate cancer, offering new perspectives for the prevention, early intervention, and treatment of prostate cancer. Based on these findings, future research should further investigate the specific biological mechanisms underlying the association between the skin microbiome and cancer, particularly how it may promote the development and progression of prostate cancer through immune and metabolic pathways. Additionally, in light of this study’s findings, future clinical research could assess the feasibility of modifying the skin microbiome to prevent or treat prostate cancer. Moreover, research should also expand to other diseases potentially influenced by the skin microbiome to explore whether similar associations exist. This could not only enhance our understanding of the skin microbiome’s crucial role in overall health but also lead to the discovery of novel biomarkers and the development of innovative therapeutic strategies.

## 6 Strengths and limitations of the study

Our study employed rigorously selected IVs to conduct a bidirectional two-sample MR analysis, investigating the relationships between 150 skin microbiota and PCa. We performed a meta-analysis of the results derived from PCa data obtained from two different sources to enhance the robustness of our findings. Additionally, we applied the Bonferroni method for multiple corrections. These methods help reduce confounding factors and reverse causality, thereby increasing the credibility of the results. However, due to the stringent experimental protocols, this approach has certain limitations and may miss some associations. It is particularly important to note that MR analysis reflects lifelong genetic susceptibility rather than short-term effects, so caution should be exercised in clinical inference. Furthermore, our genetic data are all derived from European ancestry, which may limit the applicability of our conclusions to other ancestries. Therefore, further research is needed to confirm whether these findings apply to other populations. We plan to conduct future studies involving multi-ethnic cohorts to supplement our research and further validate our conclusions.

## 7 Conclusions

The study identified a correlation between Neisseria in dry skin, one of the 150 skin microbiomes, and the risk of developing PCa, establishing it as a risk factor for increased susceptibility to PCa. However, it should be noted that this association requires further clinical validation.

## Data Availability

The original contributions presented in the study are included in the article/**Supplementary Material**. Further inquiries can be directed to the corresponding author.

## Supplementary material

Supplementary Table 1 STROBE-MR checklist.

Supplementary Table 2 Instrumental Variable Screening Results.

Supplementary Table 3 Results of the MR Analysis.

Supplementary Table 4 Results of Meta-Analysis of all Skin Microbiomes Traits.

Supplementary Table 5 Results of the Meta-Analysis.

Supplementary Table 6 Results of Cochran’s Q test.

Supplementary Table 7 Results of Horizontal pleiotropy.

Supplementary Table 8 Results of the reverse MR analysis.
